# Minisequencing mitochondrial DNA pathogenic mutations

**DOI:** 10.1186/1471-2350-9-26

**Published:** 2008-04-10

**Authors:** Vanesa Álvarez-Iglesias, Francisco Barros, Ángel Carracedo, Antonio Salas

**Affiliations:** 1 Unidade de Xenética, Instituto de Medicina Legal, Facultad de Medicina, Universidad de Santiago de Compostela, Galicia, Spain; 2 Fundación Pública Galega de Medicina Xenómica (FPGMX), Hospital Clínico Universitario, Universidad de Santiago de Compostela, Galicia, Spain

## Abstract

**Background:**

There are a number of well-known mutations responsible of common mitochondrial DNA (mtDNA) diseases. In order to overcome technical problems related to the analysis of complete mtDNA genomes, a variety of different techniques have been proposed that allow the screening of coding region pathogenic mutations.

**Methods:**

We here propose a minisequencing assay for the analysis of mtDNA mutations. In a single reaction, we interrogate a total of 25 pathogenic mutations distributed all around the whole mtDNA genome in a sample of patients suspected for mtDNA disease.

**Results:**

We have detected 11 causal homoplasmic mutations in patients suspected for Leber disease, which were further confirmed by standard automatic sequencing. Mutations m.11778G>A and m.14484T>C occur at higher frequency than expected by change in the Galician (northwest Spain) patients carrying haplogroup J lineages (Fisher's Exact test, *P*-value < 0.01). The assay performs well in mixture experiments of wild:mutant DNAs that emulate heteroplasmic conditions in mtDNA diseases.

**Conclusion:**

We here developed a minisequencing genotyping method for the screening of the most common pathogenic mtDNA mutations which is simple, fast, and low-cost. The technique is robust and reproducible and can easily be implemented in standard clinical laboratories.

## Background

There are over 100 point mutations putatively associated with human mtDNA diseases [1]; however, only a small percentage of these mutations were properly confirmed . In fact, the pathogenicity of some mutations is still under question due to the fact that the conclusions claimed in a substantial number of studies do not rest on solid grounds or are partially or completely flawed [2-5].

During this last two decades, many different techniques were designed for the screening of mtDNA pathogenic mutations, including restriction fragment length polymorphism (RFLP) analysis, heteroduplex analysis (HDA), single strand conformation polymorphisms (SSCP), etc [6-11]. The main disadvantage of these classical screening methods is that, due to the intrinsic nature of the techniques, many mutations usually pass unnoticed. Novel and more efficient strategies have recently been proposed that allow the screening of entire mtDNA genomes; for instance, the mismatch-specific DNA endonuclease "Surveyor™ Nuclease" seems to be particularly useful when the most common pathogenic mutations have been ruled out previously (e.g. [12]). Ideally, the gold standard of sequencing the whole genome would solve the problem; nevertheless, this strategy is labor-intensive and therefore unfeasible for routine molecular diagnosis (taking into account the capacities of standard laboratories). Screening methods are also difficult to standardize and some of them are not suitable for replication assays due to their dependence on for instance electrophoretic conditions.

Minisequencing techniques have demonstrated to be efficient in other fields of research, such as forensic and population genetics [13-18], but also in clinical research [19]. This approach, also known as single nucleotide primer extension, allows the determination of the polymorphic position by DNA polymerase addition of the ddNTP complementary to the base interrogated. The primer anneals therefore to its target DNA immediately adjacent to the SNP under scrutiny. Here we present a minisequencing multiplex design which is a rapid, sensitive and low cost assay for screening mtDNA pathogenic mutations in patients suffering mtDNA disease.

## Methods

### Subject and SNP selection

Blood sample of 15 patients was sent to the Fundación Pública Galega de Medicina Xenómica located in the Hospital Clínico of Santiago de Compostela (Galicia, Spain). Six of these patients are from Galicia (northwest Spain; [20]), and three other patients are from southeast Spain (in the Mediterranean coast); the regional Spanish provenance of the other two patients is unknown. Four of these patients presented clinical features of an undetermined neuropathy, while the rest of the patients were analyzed under suspicion of Leber disease. Written informed consent was obtained from all patients and the study was approved by the institutional review board of the University of Santiago de Compostela (Spain). All the samples were genotyped for a set of 25 variants, 21 are confirmed pathogenic mutations related to different mtDNA diseases such as MELAS, LHON, Leigh Disease, and NARP. These mutations are distributed all around the mtDNA molecule and 'hit' different mtDNA genes (Table 1). All the selected mutations, with the only exception of m.3243A>G, consist of non-synonymous substitutions (both transitions and transversion, but note that the minisequencing technique could be also suitable for interrogating indels [17]).

**Table 1:** Characteristics of the mutations incorporated in our multiplex design

**POSITIONS**	**Base change**	**Amino Acid change**	**Locus**	**Disease^1^**
3243	A>G	tRNA Leu (UUR)	MT-TL1	MELAS, DM/DMDT, CPEO, MM
3460	G>A	Ala>Thr	MT-ND1	LHON
3697	G>A	Gly>Arg	MT-ND1	MELAS
3946	G>A	Glu>Lys	MT-ND1	MELAS
3949	T>C	Tyr>Lys	MT-ND1	MELAS
7445	A>G	Ter>Ter	MT-CO1	SNHL
7445	A>C	Ter>Ser	MT-CO1	DEAF
8993	T>G	Leu>Arg	MT-ATP6	NARP
8993	T>C	Leu>Pro	MT-ATP6	NARP, Leigh Disease
9176	T>C	Leu>Pro	MT-ATP6	FBSN, Leigh Disease
9176	T>G	Leu>Arg	MT-ATP6	Leigh Disease
10158	T>C	Arg>Pro	MT-ND3	Leigh Disease
10191	T>C	Arg>Pro	MT-ND3	ESOC, Leigh-like Disease
10663	T>C	Val>Ala	MT-ND4	LHON
11777	C>A	Arg>Ser	MT-ND4	Leigh Disease
11778	G>A	Arg>His	MT-ND4	LHON
11832	G>A	Trp>Ter	MT-ND4	Exercise Intolerance
12706	T>C	Phe>Leu	MT-ND5	Leigh Disease
13513	G>A	Asp>Asn	MT-ND5	MELAS, Leigh Disease
13514	A>G	Asp>Gly	MT-ND5	MELAS
14459	G>A	Ala>Val	MT-ND6	LYDT, Leigh Disease
14482	C>A	Met>Ile	MT-ND6	LHON
14482	C>G	Met>Ile	MT-ND6	LHON
14484	T>C	Met>Val	MT-ND6	LHON
14487	T>C	Met>Val	MT-ND6	Dystonia, Leigh Disease

^1 ^List of some diseases where these mutations are frequently observed: CPEO: Chronic Progressive External Ophthalmoplegia; DEAF Maternally inherited DEAFness or aminoglycoside-induced DEAFness DM: Diabetes Mellitus; LDYT: Leber's hereditary optic neuropathy and DysTonia; LHON: Leber Hereditary Optic Neuropathy; MELAS: Mitochondrial Encephalomyopathy, Lactic Acidosis, and Stroke-like episodes; MM: Mitochondrial Myopathy; NARP: Neurogenic muscle weakness, Ataxia, and Retinitis Pigmentosa; alternate phenotype at this locus is reported as Leigh Disease; SNHL: SensoriNeural Hearing Loss

### Primer design

The primers both for PCR amplification (Table 2) and minisequencing reaction (Table 3) were designed to have an annealing temperature around 60°C using Primer3 software . The sequence databases at the National Centre for Biotechnology Information (NCBI; ) were interrogated using the online BLAST tool to test the primers against possible repetitive sequences and sequence homologies in the autosomal genome. Each primer pair for PCR amplification and each single base extension primer were selected independently and AutoDimer  was used to test for potential hairpin structures and primer-dimer problems.

**Table 2:** Amplification primers

**POSITIONS**	**FORWARD**	**REVERSE**	**SIZE (bp)**	**Final Concentration (μM)**
3243	tatacccacacccacccaag	ggccatgggtatgttgttaag	118	0.2
3460	ccgaacgaaaaattctaggc	gcggtgatgtagagggtgat	153	0.2
3697	gcctagccgtttactcaatcc	tgagattgtttggggctactgc	94	0.15
3946/3949^1^	tagcagagaccaaccgaacc	gaagattgtagtggtgagggtgt	157	0.2
7445	ccctaccacacattcgaagaa	tggcttgaaaccagctttg	89	0.2
8993	aatgccctagcccacttctt	aggtggcctgcagtaatgtt	140	0.15
9176	aaatcgctgtcgccttaatc	tcattaggagggctgagagg	154	0.3
10158/10191	tcaacaccctcctagcctta	gggtaaaaggagggcaattt	196	0.3
10663	acacccactccctcttagcc	ggccatatgtgttggagattg	110	0.3
11777/11778/11832	cacgggcttacatcctcatt	gggggtaaggcgaggttag	157	0.2
12706^1^	tgtagcattgttcgttacatgg	agttggaataggttgttagcgg	146	0.2
13513/13514	attggcagcctagcattagc	cagggaggtagcgatgagag	131	0.2
14459/14482/14484/14487	ctccatcgctaaccccacta	ttctgaattttgggggaggt	170	0.4

^1^These primers were reported in [16]

**Table 3:** Minisequencing primers

**POSITIONS**	**Extension Primer^1^**	**Length**	**Base change**	**Strand**	**Final Concentration (μM)^3^**
10158	acaactcaacggctacatagaaaaa	25	T>C	L	0.2
3946	Cgaactagtctcaggcttcaacatc	25	G>A	L	0.2
10663	GACTGcaatattgtgcctattgccatactag	31	T>C	L	0.2
3460	(GACT)_2_Ggctactacaacccttcgctgac	31	G>A	L	0.2
10191	(GACT)_4_agtgcggcttcgaccctata	36	T>C	L	0.3
14459	(GACT)_2_GACctcaggatactcctcaatagccatc	36	G>A	L	0.3
14484^2^	(GACT)_3_atcgctgtagtatatccaaagacaacYa	40	T>C	L	0.5
3243	(GACT)_4_GAacagggtttgttaagatggcag	40	A>G	L	0.2
11777	(GACT)_5_Gcaaactacgaacgcactcacagt	44	C>A	L	0.2
7445	(GACT)_5_tcgaagaacccgtatacataaaatctag	48	A>G/C	L	0.2
12706	(GACT)_9_gcggtaactaagattagtatggtaattagga	52	T>C	H	0.2
3949^2^	(GACT)_11_GAagtctcaggcttcaacatcRaa	52	T>C	L	0.2
14487^2^	(GACT)_7_GctgtagtatatccaaagacaaccaSca	56	T>C	L	0.6
13513	(GACT)_8_ttcctcacaggtttctactccaaa	56	G>A	L	0.1
13514	(GACT)_10_Ttgcggtttcgatgatgtgg	60	A>G	H	0.2
3697	(GACT)_9_CTaaactcaaactacgccctgatc	60	G>A	L	0.2
11778	(GACT)_9_GAgaagtccttgagagaggattatgatg	64	G>A	H	0.3
11832	(GACT)_8 _GACtcaaactctactcccactaatagcttttt	64	G>A	L	0.1
9176	(GACT)_11_Gatccaagcctacgttttcacacttc	70	T>C/G	L	0.2
14482	(GACT)_11_gccatcgtcgctgtagtatatccaaagacaac	73	C>A/G	L	0.6
8993	(GACT)_13_Gcctactcattcaaccaatagccc	76	T>G/C	L	0.2

^1 ^Capital letters indicate the segment of the primer belonging to the tail

^2 ^These primers are 'degenerated' at the indicated position in the second column (see Material and Methods) using the IUB code

Although the simultaneous occurrence of more than one pathogenic mutation in a single individual is infrequent, it could be possible that the presence of m.14484T>C could interfere with the genotyping of m.14487T>G (due to their physical proximity). Therefore, in order to avoid potential artifacts due to deficient annealing, we have designed a degenerate extension primer containing a mixture of nucleotide C and T at site 14484. The same rationale applies to variation at other two positions. According to our design, m.14482C>G mutation could interfere with the genotyping at position 14484; and the presence of m.3946G>A could alter the genotyping of m.3949T>C; we therefore designed another extension degenerate primer containing C and G at site 14482 and another additional extension degenerated primer carrying G and A at site 3946 (Table 3).

### PCR multiplex amplification

The SNPs were PCR amplified in 13 amplicons with sizes ranging from 89 and 196 bp. The amplicons are deliberately designed to be small in order to facilitate the analysis of samples that are highly degraded or with low quantity of DNA. Some amplicons encompass several SNPs (Table 1). We performed multiplex reaction using 5 ng of DNA template and PCR master mix of QIAGEN Kit Multiplex PCR (Qiagen, Hilden, Germany), amplification primers and their final concentrations are in Table 2. Amplification was carried out in a 9700 Thermocycler (Applied Biosystems, Foster City, CA, USA). After a 95°C pre-incubation step for 15 minutes, PCR was performed in a total of 30 cycles using the following conditions: 94°C denaturation for 30 seconds, annealing at 60°C for 90 seconds and extension at 72°C during 90 seconds, followed by a 15 minutes of final extension at 72°C and 4°C until removed from thermocycler. PCR products were checked by polyacrylamide gel electrophoresis (T9, C5) visualized by silver staining.

### Minisequencing reaction

Previous to minisequening reaction, PCR products are treated with ExoSAP-IT (Amershan Bioscences, Uppsala, Sweden) to remove excess primers and un-incorporated dNTPs: 3 μl of PCR product was incubated with 1.5 μl for 15 minutes at 37°C followed by 15 minutes at 80°C for enzyme inactivation. Minisequencing reaction is performed using SNaPshot™ Kit (AB). We modified the length of the primers (between 25 and 76 bp) by the addition of non-homologous tails, poly(dGACT) added at the 5'- end (Table 3). The minisequencing reaction was carried out in a total volume of 10 μl comprising 3 μl of the SNaPshot™ Kit (AB), 1.5 μl PCR product, 1.5 μl of extension primers mix (final concentrations are between 0.1 and 0.6 μM) (Table 3), and water up to 10 μl. The reaction was performed in a 9700 Thermocycler (AB) following the recommendations of the manufacturer: 25 cycles of denaturation at 96°C for 10 seconds, annealing at 50°C for 5 seconds and extension at 60°C during 30 seconds. Un-incorporated ddNTPs are eliminated with a treatment with SAP (Amershan Bioscences). The final volume (10 μl) was treated with 1 μl of SAP for 60 minutes at 37°C followed by 15 minutes at 80°C for enzyme inactivation.

The minisequencing products (1.5 μl) were mixed with 10 μl of HiDi™ formamide (AB) and 0.25 μl of GeneScan -120 LIZ (AB) and capillary electrophoresis was undertaken on an ABI PRISM 3130 ×l Genetic Analyzer (AB). The data was analyzed using GeneMapper™ 3.7 Software (AB).

### Sequencing reaction

All the 13 amplicons were sequenced in an ABI PRISM 3130 ×l Genetic Analyzer (AB) for all the patients with the aim of corroborating the mutations observed with the minisequencing assay. Technical details regarding sequencing reaction are given in [17].

## Results

Only 13 amplicons are needed to interrogate the selected 25 variants. This is because (i) 'neighbouring' variants are genotyped using different minisequencing probes annealing at the same amplicon, and (ii) different minisequencing probes were designed to genotype different pathogenic substitutions occurring at the same mtDNA site. Therefore, for each of the sites 7445, 9176, and 14482, there are two common mtDNA mutations described in the literature as pathogenic, the most common one for each of these three sites was already confirmed (m.7445A>G, m.9176T>C, and m.14482C>G), while the alternative one (m.7445A>C, m.9176T>G, and m.14482C>A) still bears the status of 'provisional' in MITOMAP. In addition, position 8993 has two already confirmed variants, namely, T to C and T to G substitutions.

**Figure 1:**
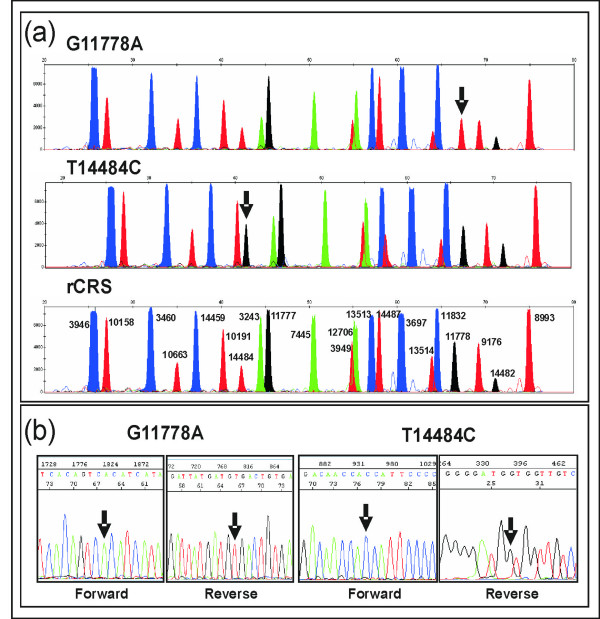
Electropherograms showing (a) two different SNaPshot profiles carrying mutations m.11778G>A and m.14484T>C; rCRS [50] electropherogram is also shown indicating the whole set of mutations tested with the SNaPshot reaction; and (b) forward and reverse sequence electropherograms for these same mutations.

We did not observe mutations in four patients presenting neurological symptoms. On the other hand, a pathogenic mutation was found for each of the other 11 patients with clinical suspicion of Leber disease (Table 4). All the mutations were confirmed by sequencing and were observed as homoplasmic using both minisequencing and automatic sequencing (Figure 1). Nine patients carried mutation m.11778G>A whereas two patients carried m.14484T>C.

**Table 4:** Patient samples analyzed in the present study

		**3**	**3**	**3**	**3**	**3**	**7**	**8**	**9**	**1**	**1**	**1**	**1**	**1**	**1**	**1**	**1**	**1**	**1**	**1**	**1**	**1**	
		**2**	**4**	**6**	**9**	**9**	**4**	**9**	**1**	**0**	**0**	**0**	**1**	**1**	**1**	**2**	**3**	**3**	**4**	**4**	**4**	**4**	
		**4**	**6**	**9**	**4**	**4**	**4**	**9**	**7**	**1**	**1**	**6**	**7**	**7**	**8**	**7**	**5**	**5**	**4**	**4**	**4**	**4**	
		**3**	**0**	**7**	**6**	**9**	**5**	**3**	**6**	**5**	**9**	**6**	**7**	**7**	**3**	**0**	**1**	**1**	**5**	**8**	**8**	**8**	
										**8**	**1**	**3**	**7**	**8**	**2**	**6**	**3**	**4**	**9**	**2**	**4**	**7**	**Variants^1^**
	**rCRS**	**A**	**G**	**G**	**G**	**T**	**A**	**T**	**T**	**T**	**T**	**T**	**C**	**G**	**G**	**T**	**G**	**A**	**G**	**T**	**T**	**T**	
#1	I-111	-	-	-	-	-	-	-	-	-	-	-	-	**A**	-	-	-	-	-	-	-	-	
#2	D-112	-	-	-	-	-	-	-	-	-	-	-	-	**A**	-	-	-	-	-	-	-	-	11812
#3	I-138	-	-	-	-	-	-	-	-	-	-	-	-	**A**	-	-	-	-	-	-	-	-	10172
#4	F-393	-	-	-	-	-	-	-	-	-	-	-	-	**A**	-	-	-	-	-	-	-	-	
#5	C-436	-	-	-	-	-	-	-	-	-	-	-	-	-	-	-	-	-	-	-	-	-	
#6	B-703	-	-	-	-	-	-	-	-	-	-	-	-	-	-	-	-	-	-	-	-	-	
#7	B-900	-	-	-	-	-	-	-	-	-	-	-	-	**A**	-	-	-	-	-	-	-	-	
#8	B-901	-	-	-	-	-	-	-	-	-	-	-	-	**A**	-	-	-	-	-	-	-	-	
#9	D-992	-	-	-	-	-	-	-	-	-	-	-	-	-	-	-	-	-	-	-	-	-	
#10	1586	-	-	-	-	-	-	-	-	-	-	-	-	**A**	-	-	-	-	-	-	-	-	
#11	5699	-	-	-	-	-	-	-	-	-	-	-	-	-	-	-	-	-	-	-	**C**	-	12696
#12	5869	-	-	-	-	-	-	-	-	-	-	-	-	-	-	-	-	-	-	-	**C**	-	12696
#13	G-495	-	-	-	-	-	-	-	-	-	-		-	**A**	-	-	-	-	-	-	-	-	
#14	7218	-	-	-	-	-	-	-	-	-	-	-	-	-	-	-	-	-	-	-	-	-	
#15	9615	-	-	-	-	-	-	-	-	-	-	-	-	**A**	-	-	-	-	-	-	-	-	12705C/T

^1 ^Variants detected by automatic sequencing (putatively unrelated to the disease outcome). Position 12705 is heteroplasmic C/T.

The two carriers of m.14484T>C lived in the same geographic (relatively isolated) village (namely, Burela) in the northern cost of Galicia [20,21]. These samples were submitted at different times to the laboratory and according to the carriers, their families were unrelated. Both patients share also the transition m.12696T>C (observed by sequencing), indicating that these individuals could actually share some recent common ancestor. The m.12696T>C transition is not a frequent variant in Europe and likely constitutes a diagnostic variant of a minor HV1 sub-lineage (although this variant is also recurrent in at least other eight non-European haplogroups, including haplogroup J, see below): it appears in at least six haplogroup HV1 complete genomes (see for instance [22,23]). Therefore, the m.14484T>C transition could represent a founder pathogenic mutation responsible of Leber disease in Galicia (as it is the case of a wide spectrum of different non-mtDNA diseases [24,25].

In order to further investigate this hypothesis, we additionally genotyped all the Galician samples for a set of haplogroup diagnostic mtDNA SNPs following [16] (Table 5). The two carriers of m.14484T>C but also two other Galician samples carrying m.11778G>A could be allocated to haplogroup J (and not to HV1 as it could be inferred by the presence of m.12696T>C). Therefore, the observation of the m.14484T>C mutation in two unrelated patients from the above mentioned small Galician village could just reflect the already reported high incidence of Leber mutations within haplogroup J [26,27]. In fact, we also observe that the frequency of haplogroup J in healthy unrelated Galician individuals is ~14.7% [20,21,28] but it occurs in four out of our six Galician patients (~66.7%); the difference in prevalence is statistically significant (Fisher's Exact test, *P*-value < 0.006)

**Table 5:** Coding SNP and haplogroup status of the Galician patients

											**1**	**1**	**1**	**1**	**1**	**1**	**1**	**1**	
		**3**	**3**	**4**	**4**	**4**	**4**	**4**	**6**	**7**	**0**	**0**	**0**	**0**	**2**	**2**	**3**	**4**	
		**9**	**9**	**2**	**5**	**5**	**7**	**7**	**7**	**0**	**3**	**4**	**4**	**8**	**3**	**7**	**9**	**7**	
		**1**	**9**	**1**	**2**	**8**	**6**	**9**	**7**	**2**	**9**	**0**	**6**	**7**	**0**	**0**	**6**	**6**	
		**5**	**2**	**6**	**9**	**0**	**9**	**3**	**6**	**8**	**8**	**0**	**3**	**3**	**8**	**5**	**6**	**6**	**HG**
	**rCRS**	**G**	**C**	**T**	**A**	**G**	**A**	**A**	**T**	**C**	**A**	**C**	**T**	**T**	**A**	**C**	**A**	**C**	
#3	I-138	**-**	**-**	**C**	**-**	**-**	**-**	**-**	**-**	**T**	**G**	**-**	**-**	**-**	**-**	**-**	**-**	**T**	J
#4	F-393	**-**	**-**	**C**	**-**	**-**	**-**	**-**	**-**	**T**	**G**	**-**	**-**	**-**	**-**	**-**	**-**	**T**	J
#10	1586	**-**	**-**	**-**	**-**	**-**	**-**	**-**	**G**	**-**	**-**	**-**	**-**	**-**	**-**	**-**	**-**	**-**	H3
#11	5699	**-**	**-**	**C**	**-**	**-**	**-**	**-**	**-**	**T**	**G**	**-**	**-**	**-**	**-**	**-**	**-**	**T**	J
#12	5869	**-**	**-**	**C**	**-**	**-**	**-**	**-**	**-**	**T**	**G**	**-**	**-**	**-**	**-**	**-**	**-**	**T**	J
#13	G-495	**-**	**-**	**-**	**-**	**-**	**-**	**-**	**-**	**T**	**-**	**-**	**-**	**-**	**G**	**-**	**-**	**T**	U

HG = haplogroup

Other variants were also observed by sequencing analysis in m.11778G>A carriers. For instance, the transition A11812G is diagnostic of haplogroup T2 and G10172A identifies haplogroup J2b; these variants also appear sporadically in other haplogroup backgrounds. In addition, we observe a heteroplasmy at position 12705; transition C12705T is a well-known diagnostic site that (together with T16223C) leads from macro-haplogroup N to R.

**Figure 2:**
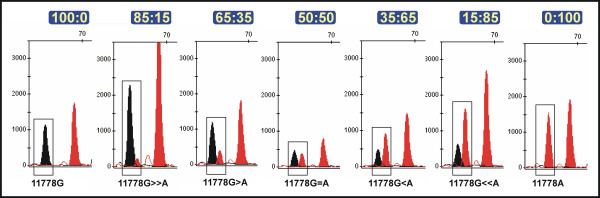
Partial electropherograms showing the performance of the SNaPshot assay with mixtures of wild:mutant DNAs. Numbers in the top indicate the percentages of wild:mutant mixture.

Mixtures of two different DNA bearing different SNP profiles were carried out in order to simulate mtDNA heteroplasmy, a common state in mtDNA disease patients. As shown in Figure 2, the minisequencing assay is perfectly able to detect the mixtures at different proportions. According to [29] "*the single most important disadvantage of the SNaPshot is that it seems less accurate than conventional PCR-RFLP analysis for *[detecting] *high levels of mutant mtDNA*". Our results however indicate that the ability of SNaPshot for detecting heteroplasmy is actually equivalent to standard sequencing. Some fluorocromes 'project' more intensity in the electropherogram than other (as it also occurs with some sequencing chemistries), and this should be taking into account when quantifying the real proportions of wild:mutant DNA (heteroplasmy).

Although our primer design take into account the proximity of two pathologic mutations, it is not possible to consider all the potential polymorphisms that could hit within the annealing segments, and therefore alter the PCR efficacy (and in the worst of the cases, produce an artefact related to e.g. the spurious amplification of a NUMT; see below). This is however an universal problem affecting most of the genotyping techniques (including RFLP and sequencing) and therefore, the expert should be always alert under the possibility of false negative results. The experience of previous SNaPshot designs [16,18,30] indicates however that the method is robust under the presence of polymorphisms.

Finally, the multiplex reaction was also tested in 102 control individuals. There were not false positives in this control sample and the SNP-minisequencing profiles were phylogenetically concordant [31,32].

## Discussion

MtDNA mutations are responsible for various clinical features, which often make diagnosis a considerable challenge. Since the mitochondria constitute the energetic 'factory' of all nucleated cells, mtDNA diseases affect many tissues with variable clinical outcomes. When searching the Mitomap database for pathogenic mtDNA mutations, many of them harbour the status of 'provisional', while some other stated as 'confirmed'; among the later, some were recently questioned [2,33,34]. We here selected a total of 25 mutations, 21 of them were confirmed as pathogenic in common mtDNA diseases in multiple independent studies and considering different criteria, including the score system proposed by [33] (see also [35]) and also the complementary phylogenetic approach [2]. For instance, we included all the MTND gene mutations evaluated with the complex I pathogenicity scoring system of [33] (see their Table 2) and reaching the 16 top values (≥ 30). Apart from the 21 confirmed mutations included in the design, the remaining four mutations were additionally included because they occur at lower frequencies at four of the initially 21 selected positions.

Among the selected mutations, we have the transition m.3243A>G which is a typical cause of MELAS [36] and MIDD [37]; transition m.8993T>G, responsible of a number of patients suffering NARP [38] and MILS [39]; transitions m.3460G>A, m.11778G>A, m.14484T>C, which are common causal mutations in LHON patients [40,41], etc. (see [42] for a review). It can be tentatively said that the selected SNPs actually cover an ample spectra of the fully confirmed causal mutations in the most common mtDNA diseases. For instance, according to [43], the prevalence of m.3243A>G is about 1 in 6,135 in the general (European) population; see also [44]. It is also worth to mention that it is straightforward to add mutations to our initial multiplex design because SNaPshot minisequencing is very flexible in this regard [17].

## Conclusion

The minisequencing reaction presented here detected 11 mutations in an easy and straightforward manner in 11 different patients with clinical suspicion of Leber disease. All the mutations and their homoplasmic status were finally confirmed by automatic sequencing. We did not observed false positives in a substantial number of controls. We also simulated heteroplasmic states by mixing wild:mutant DNAs; the minisequencing technique demonstrates a good performance in detecting at least mixtures up to 1:10.

The multiplex reaction shows various advantages in the clinical field with respect to other techniques (see [17] for more details). For instance, the primers were designed in order to obtain amplicons of size ranging 89–196, a feature of special interest in a clinical context where often the laboratories have to deal with suboptimum samples containing low amounts of DNA or highly degraded DNA (paraphine imbibed samples, biopsies, etc.) as it is also the case with forensic samples [13]. Finally, we estimate that the cost of genotyping 25 SNPs is at least 20 times lower than the cost of RFLP genotyping (considering genotyping of one mutation at the time), and it can be done in less than three hours (including post-PCR purification, minisequencing reaction, purification of minisequencing products, electrophoresis, and documentation) for as many samples as the number of capillaries of the automatic sequencer.

Also important is the fact that minisequencing is robust respect potential artefacts related to false positives at NUMTs (author's unpublished data). Multiplex genotyping also prevents from artificial recombination; the later is a kind of common artefact that more easily arise as more independent fragments are genotyped for the same sample [31,32,45-49].

Finally, the screening method presented here should not be considered as a substitute of complete genome sequencing. The latter should be performed when the minisequencing screening (or the use of other alternative strategies) fails to identify the causal mutation.

## Competing interests

The author(s) declare that they have no competing interests.

## Authors' contributions

VAI, FB, AC, and AS designed the study, collected and analyzed the data, and wrote the paper.

## Availability and requirements

There are over 100 point mutations putatively associated with human mtDNA diseases [1]; however, only a small percentage of these mutations were properly confirmed . The primers both for PCR amplification (Table 2) and minisequencing reaction (Table 3) were designed to have an annealing temperature around 60°C using Primer3 software . The sequence databases at the National Centre for Biotechnology Information (NCBI; ) were interrogated using the online BLAST tool to test the primers against possible repetitive sequences and sequence homologies in the autosomal genome. Each primer pair for PCR amplification and each single base extension primer were selected independently and AutoDimer  was used to test for potential hairpin structures and primer-dimer problems.

## Pre-publication history

The pre-publication history for this paper can be accessed here:


